# Triple M Syndrome Associated With Concurrent Durvalumab and Olaparib Therapy in Metastatic Cholangiocarcinoma: A Case Report

**DOI:** 10.1155/crom/1318038

**Published:** 2026-06-22

**Authors:** Oliver Nilsen, Clare Senko, Allison Collins, Nicola Tomkins, Daniel Buckley, Andrew Weickhardt, Niall Tebbutt

**Affiliations:** ^1^ Department of Medical Oncology, Austin Health, Heidelberg, Victoria, Australia, austin.org.au; ^2^ Olivia Newton-John Cancer Wellness and Research Centre, Austin Health, Heidelberg, Victoria, Australia, austin.org.au; ^3^ School of Cancer Medicine, La Trobe University, Bundoora, Victoria, Australia, latrobe.edu.au; ^4^ The University of Melbourne, Austin Health, Heidelberg, Victoria, Australia, austin.org.au

**Keywords:** case report, durvalumab, immune checkpoint inhibitor, olaparib, PARPi, triple M syndrome

## Abstract

Triple M syndrome is a rare and life‐threatening overlap presentation of myocarditis, myositis and myasthenia gravis, secondary to immune checkpoint inhibition. We report a case of an 80‐year‐old female with metastatic cholangiocarcinoma, presenting with progressive asthenia, ptosis and dysphagia, following completion of six cycles of combination chemotherapy and immunotherapy, durvalumab. Her symptoms emerged 2 weeks after the commencement of maintenance therapy with immune checkpoint inhibitor, durvalumab, and poly(ADP‐ribose) polymerase inhibitor, olaparib. Biochemical investigations were suspicious for myocarditis, myositis and type 2 respiratory failure due to myasthenic crisis. Clinical findings supported the diagnosis of triple M syndrome, and she was treated with high‐dose steroids, intravenous immunoglobulin and supported with non‐invasive ventilation. The patient initially improved; however, her admission was complicated by a fatal retroperitoneal haemorrhage. Our case highlights the need to consider serious immune‐related adverse events and escalate prompt management for overlap syndromes in the context of immunotherapy and poly(ADP‐ribose) polymerase inhibitors.

**Trial Registration:**

ClinicalTrials.gov identifier: NCT06441747

## 1. Introduction

Cholangiocarcinoma is an aggressive malignancy arising from the epithelial lining of the bile ducts and accounts for only a small proportion of gastrointestinal cancers [1]. The mainstay systemic therapy has been a combination of platinum‐based chemotherapy, including cisplatin and gemcitabine. In recent years, advances in systemic therapy have incorporated immune checkpoint inhibitors (ICIs) to standard of care chemotherapy. Specifically, the TOPAZ‐1 Phase III trial [2] demonstrated that by adding durvalumab, a monoclonal antibody targeted against programmed death‐ligand 1 (PD‐L1), to cisplatin and gemcitabine, this significantly improved overall survival in patients with advanced biliary malignancies.

The immune‐related adverse events (irAEs) associated with ICI therapy are well‐recognised complications, which can affect any organ system. The frequency of irAEs depends on the agents used and specific patient characteristics [3]. Common irAEs include cutaneous reactions, colitis and endocrinopathies. However, cardiac and neurological irAEs are far less common and can be severe and life‐threatening when they arise.

A particularly serious manifestation of irAE includes the overlap of myocarditis, myositis and myasthenia gravis, termed triple M syndrome [4]. The concurrent presentation of these three conditions is observed in only 0.1%–0.3% of patients undergoing ICI therapy [5]. Individually, they confer a high‐mortality risk; however, presenting together, triple M syndrome in‐hospital mortality rates approach 60% [5, 6]. Myocarditis, myositis and myasthenia gravis may share overlapping features, which makes the diagnosis challenging. The true incidence of triple M syndrome is unknown; however, there are multiple published case reports and case series following ICI‐associated triple M syndrome [7–14]. Most published cases have been associated with anti‐PD‐1 therapy, including nivolumab and pembrolizumab, with fewer cases describing triple M syndrome in the context of anti–PD‐L1, such as durvalumab [7, 12]. Additionally, the combination of poly(ADP‐ribose) polymerase inhibitor (PARPi), such as olaparib, with ICI may enhance antitumour effectiveness [15, 16], thereby potentially predisposing to higher rates of irAE.

Here, we report a fatal case of triple M syndrome in an elderly woman receiving durvalumab and olaparib treatment for metastatic cholangiocarcinoma. This case illustrates the importance of considering rare and overlapping irAEs and emphasises the importance of implementing a multidisciplinary approach to manage immunotherapy‐related toxicity. To our knowledge, this case adds to the reports of triple M syndrome in the setting of durvalumab and is the only triple M syndrome case report of coupled ICI and PARPi therapy.

## 2. Case Presentation

The patient was an 80‐year‐old woman with metastatic cholangiocarcinoma. She experienced triple M syndrome in the setting of durvalumab (anti–PD‐L1) treatment for metastatic cholangiocarcinoma, with metastatic sites of disease being portacaval mass and omental disease. The patient′s cholangiocarcinoma was diagnosed following a previous admission for cholecystitis, where she underwent laparoscopic cholecystectomy. This is on a background of chronic cholelithiasis and intraductal papillary mucinous neoplasms, which were being monitored annually. She had already completed six cycles of first‐line therapy with cisplatin, gemcitabine and durvalumab, with an upfront 20% chemotherapy dose reduction. Just prior to her admission, she was consented to a clinical trial, which incorporated maintenance 1,500 mg intravenous 4‐weekly durvalumab for 2 years and continuous 300 mg olaparib BD. She received only one cycle of maintenance durvalumab, representing her seventh overall dose of durvalumab. Her baseline ECOG performance status was one.

Her medical issues included type 2 diabetes mellitus managed with metformin 1000 mg BD, hypertension but not on antihypertensive agents, osteoporosis managed with alendronate 70 mg PO weekly and vitamin D 1,000 IU PO daily, polymyalgia rheumatica managed with 2 mg PO prednisolone daily and Panadol osteo PO 1,330 mg TDS and depression and anxiety managed with sertraline 50 mg PO daily and olanzapine 2.5 mg nocte. She had a history of pulmonary embolism managed with 2.5 mg apixaban BD.

She reported significant fatigue and dramatic decline in function 2 weeks after the first olaparib dose and seventh dose of durvalumab. The olaparib was withheld during the 4th‐week cycle with no improvement. She then presented to hospital due to grade 3 aspartate transaminase (AST) and alanine transaminase (ALT) elevation, presumed to be an irAE hepatitis. However, she had a normal liver ultrasound and liver screen shortly prior to admission.

On admission, day one, she recounted 2 weeks of progressive fatigue, lethargy and anorexia. This was associated with weight loss in the weeks preceding admission. Additionally, she experienced exertional dyspnoea one week prior to admission and was dysarthric and dysphagic with both solids and liquids. Her physical examination revealed pronounced left ptosis and moderate right ptosis. There was no overt ophthalmoplegia or diplopia at rest. However, there was evidence of horizontal diplopia on left gaze. Her left ptosis showed evidence of fatiguability with prolonged upward gaze. Additionally, she had bilateral neck extensor and jaw weakness, both 4/5 power. Her ankle dorsiflexion and plantar flexion were 5/5 power, and wrist and finger flexion and extension were 5/5 bilaterally. The remainder of her upper and lower limb power was 4+/5. There was no evidence of ataxia, and fine touch, temperature and vibration sensation were intact. Her reflexes were difficult to elicit.

Her day two biochemistry profile showed significantly raised creatinine kinase (CK) 1,934 units/L (normal value between 30–150) and troponin I 200 ng/L (normal value < 10). Acetylcholine receptor antibody (aChR) and anti‐muscle‐specific kinase (MUSK) were both negative. She had a rise in both ALT, 324, and AST, 269, with normal bilirubin. Her initial electrocardiogram (ECG) demonstrated T wave inversion in V2/V3 and flattened T waves. On day two, her best forced vital capacity (FVC) was 0.74 L, and her oxygen saturations were 85% on room air. Her blood gas analyses demonstrated acidosis with pH 7.29 and hypercapnia, consistent with type 2 respiratory failure. A CT brain and neck did not demonstrate any abnormalities. In view of her respiratory compromise and presumed presentation of myasthenic crisis, she was transferred to the intensive care unit (ICU) for monitoring and commenced non‐invasive ventilation (NIV). Given her known metastatic malignancy and co‐morbid status, she was not for invasive ventilation.

On day two of her admission, she was commenced on 1,000 mg intravenous methylprednisolone for 72 hours. She then switched to an equivalent dosing of prednisolone (1 mg/kg), including intravenous dexamethasone 9 mg when nil by mouth. In consultation with neurology, she was commenced on intravenous immunoglobulin (IVIG), 2 g/kg, in five daily doses totaling 115 g of IVIG. She was also commenced on 30 mg pyridostigmine TDS. In the event of further deterioration, two rescue medications, abatacept and ruxolitinib, were for salvage treatment. Nerve conduction studies and electromyography were planned but did not eventuate.

**Figure 1:**
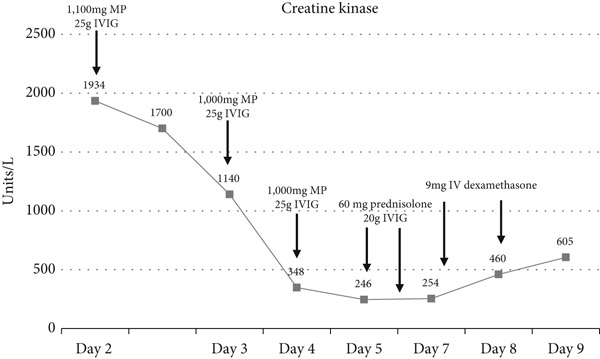
Creatinine kinase levels throughout admission. IVIG: intravenous immunoglobulin; MP: methylprednisolone.

**Figure 2:**
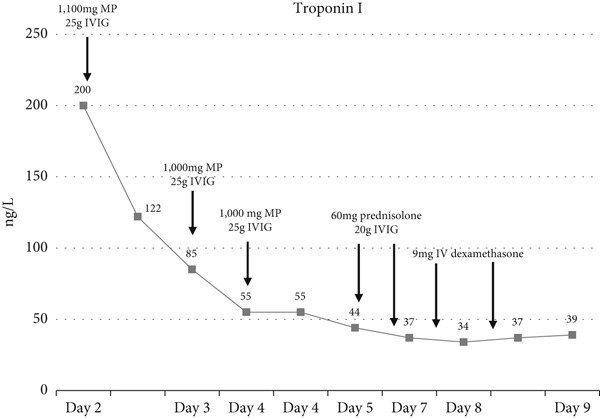
Troponin I levels throughout admission. IVIG: intravenous immunoglobulin; MP: methylprednisolone.

She initially improved with a combination of IVIG and high‐dose steroids, with a decline in serial CK (Figure 1) and troponin I (Figure 2). Her serial FVCs also improved. Alongside cardiology input, she was commenced on an ACE inhibitor. She was planned to commence a low‐dose beta blocker for myocarditis, once improved from the myasthenic crisis; however, this was never initiated during her admission. She had an inpatient transthoracic echocardiogram, which showed overall low‐normal left ventricular (LV) systolic function, LV ejection fraction of 52%–55%, an enlarged left atrium and valvular functions within normal limits. There was no pericardial effusion. She was planned for cardiac magnetic resonance imaging. She was transferred from the ICU to the ward on day five of admission, with ongoing NIV support. An MRI brain on day eight was negative for abnormalities.

On day nine, she developed hypotension and haemoglobin (Hb) drop from 112 g/L to 72 g/L over a period of 48 hours. She had a CT angiogram that demonstrated a large left retroperitoneal bleed, and she was subsequently transferred back to ICU. Her Hb decreased to a trough of 53 g/L. There was no immediately identifiable arterial site of origin. Due to her nil by mouth status, she had been switched from 2.5 mg apixaban BD to therapeutic enoxaparin 60 mg BD during her admission. The patient′s large retroperitoneal bleed was thought to be secondary to this therapeutic anticoagulation. The retroperitoneal bleed was not amenable to surgical or radiological intervention. Her anti‐Xa level was 0.67 units/mL at this time, and her coagulation studies were otherwise normal. Attempts to reverse bleeding were initiated, including pack red blood cell transfusions, fresh frozen plasma and protamine administration. Upon attempting to repeat a CT angiogram to ascertain any potential targets amenable to interventional radiology, the patient suffered a cardiac arrest with pulseless electrical activity and died on day ten of admission.

## 3. Discussion

This case highlights the emerging triple M syndrome associated with a triad of myocarditis, myositis and myasthenia gravis secondary to ICI. Although each of these three syndromes can occur individually, their overlap is infrequent. To our knowledge, this case adds to one of the few existing triple M syndrome case reports in the literature [7–12, 14, 17], for which a minority of cases discuss durvalumab‐induced triple M syndrome [7, 12]. Currently, this case is the only triple M syndrome report outlining concurrent maintenance durvalumab and olaparib treatment in the context of metastatic cholangiocarcinoma.

A notable feature is the late onset of irAE‐associated symptoms, occurring 8 months following the initiation of chemotherapy and durvalumab (six cycles) and two weeks post maintenance durvalumab (Cycle 1), without concurrent chemotherapy. Many irAEs present early following ICI [18], although they can occur at any timepoint, and the presence of irAE is associated with improved progression‐free survival [19, 20]. Additionally, the commencement of PARPi, olaparib, weeks prior to our patient′s admission may have also contributed to her clinical presentation and severe irAE overlap manifestation.

Interestingly, a recent case series of 19 patients with ICI‐mediated myasthenia gravis and overlap syndrome noted that none were treated with concurrent chemotherapy [21]. This case aligns with that observation, such that our triple M syndrome presentation emerged following the commencement of maintenance durvalumab, after six cycles of prior chemoimmunotherapy exposure. However, the current literature does not support the concept that concurrent chemotherapy harbours a protective effect for severe ICI‐mediated toxicity or overlap syndromes. Typically, combination chemoimmunotherapy is associated with an increased incidence of adverse events [22, 23] and hence, further insight into elucidating this relationship may be warranted.

### 3.1. Immunotherapy and PARPi

Most triple M syndrome case reports are associated with anti–PD‐1 therapy, such as nivolumab and pembrolizumab. Anti–PD‐L1 agents, including durvalumab, have comparatively fewer reports. Our patient was receiving PARPi, olaparib, which can demonstrate robust antitumour effects in BRCA1/2 mutant solid tumours [24, 25]. In preclinical murine models, the addition of PARPi to ICI has been suggested to generate a synergistic activity, increasing tumour immunogenicity and promoting CD8+ T cytotoxic cell tumour infiltration via the STING pathway [26]. Furthermore, the effectiveness of the olaparib and durvalumab combination is supported by the MEDIOLA trial [27], which demonstrated promising antitumour activity and safety profiles in 34 enrolled patients compared with monotherapy alone. Understandably, with augmented ICI activity, more severe irAEs might be expected. However, previous trials have suggested that the combination of PARPi and ICI demonstrates a similar incidence of any grade and grade 3+ irAEs [28]. Interestingly, the combination of ICI and PARPi may predispose patients to higher rates of irAE hepatitis, as suggested by elevated AST/ALT [29], which was observed in our patient. Therefore, the interaction of olaparib and durvalumab may have contributed to the presentation of triple M syndrome. Hence, caution should be used in elderly patients with comorbidities and frailty who may be at greater risk for enhanced immune toxicity and adverse events with the ICI and PARPi combination.

### 3.2. Diagnostic Challenge

Triple M syndrome remains a diagnostic dilemma due to the symptom overlap and often subacute presentation. Our patient′s chief complaint centred around generalised weakness and ptosis, with the development of biochemical derangements in CK and troponin suggesting muscle and cardiac inflammation. Recognition of clinical hints for myositis remains crucial and may be most obvious initially. An important point from this case is that the patient′s elevation in AST and ALT may have reflected evolving myositis, rather than hepatitis. The distinction is crucial since misinterpretation may delay accurate diagnosis and management of triple M syndrome. This is particularly true in the early stages of triple M syndrome, where myositis and myocarditis may precede overt neuromuscular symptoms. Hence, clinicians should interpret biochemical transaminase elevations in the context of CK, troponin and clinical presentation, maintaining an index of suspicion for myositis.

The suggestion of myositis should encourage clinicians to investigate for myocarditis, which may not present with chest pain, and is diagnosed in up to 30% of patients with immune‐related myositis [30]. Myocarditis can be rapidly fatal, especially in the context of abrupt arrhythmia. Conversely, concurrent myocarditis occurs in approximately 8% of cases with immune‐related myasthenia gravis [31], which suggests that it may be under‐recognised but clinically significant. Additionally, the diagnosis of immune‐related myasthenia gravis often relies on clinical assessment. Acetylcholine receptor and anti‐MuSK antibodies were negative in this case, which highlights that seronegativity does not necessarily exclude triple M syndrome diagnosis [32]. Up to two‐thirds of patients with irAE myasthenia gravis have positive antiacetylcholine receptor antibodies, which tends to be lower than that of idiopathic myasthenia gravis [33]. Hence, autoantibodies may not be helpful in diagnosing acute triple M syndrome, especially given the turnaround time for testing, balanced against the acute urgency for treatment. This is also true for neurophysiological investigation, which would ordinarily aid with diagnosing the myasthenia gravis component, but often time is a restrictive factor. Overall, the triad overlap highlights the utmost importance of maintaining high clinical suspicion, as early identification of biochemical abnormalities and subtle symptomatology proves critical to mitigating substantial mortality risk.

### 3.3. Management Considerations

Currently, the management for triple M syndrome is broadly extrapolated from treatment paradigms for isolated irAEs and guidelines such as The European Society for Medical Oncology [34]. Our case supports key themes that have been previously described in suggested triple M syndrome management strategies, namely the importance of early recognition and prompt initiation of immunosuppressive agents to improve outcomes [35]. High‐dose corticosteroids, including intravenous methylprednisolone, remain the cornerstone recommendations in severe ICI toxicity. IVIG or plasma exchange is often necessary to address acute neuromuscular weakness [6], secondary to the myasthenia component. Patients with ICI‐induced myasthenia gravis tend to do better with early implementation of either IVIG or plasma exchange, alongside corticosteroids [36]. Beyond corticosteroids and IVIG, second‐line treatment recommendations differ. These include the use of nonsteroidal immunosuppressive agents ruxolitinib, a JAK inhibitor, and abatacept, a CTLA‐4 fusion protein, in ICI‐myocarditis [37, 38]. This extends to a recent case report of refractory triple M syndrome [12], whereby ruxolitinib and abatacept were implemented effectively. Though our patient initially improved with corticosteroids and IVIG, both abatacept and ruxolitinib were considered as rescue medications. The use of other steroid‐sparing, immunosuppressive agents, which incorporate mycophenolate mofetil, azathioprine and biologics like rituximab, could also be considered and have been reported in the literature [4].

Nevertheless, there remain no dedicated guidelines for the management of complex overlap irAEs. Gradually, triple M syndrome is being recognised and described, which warrants further development of evidence‐based guidelines to support treating clinicians with this complex presentation. As the use of ICIs expand to more tumour types and older populations, recognition of uncommon and potentially fatal irAEs will be critical. Prospective data are still needed to identify risk factors and biomarkers that could help clinicians determine ICI response and toxicity risk.

## 4. Conclusion

Triple M syndrome is a rare and emerging entity amongst patients receiving ICI therapy. This case highlights the first reported triple M syndrome with concurrent ICI and PARPi therapy in metastatic cholangiocarcinoma. Clinicians should be increasingly vigilant of serious irAE overlap syndromes, including in the context of combination ICI and PARPi treatment. Improving our collective understanding of triple M syndrome, and emerging overlap irAE phenomena, is critical to augment recognition, multidisciplinary management and patient outcomes.

## Consent

Written informed consent for publication was obtained from the patient′s next of kin. All data have been anonymized prior to publication to protect the identity of the patient.

## Conflicts of Interest

The authors declare no conflicts of interest.

## Funding

No funding was received for this manuscript.

## Data Availability

Data sharing not applicable to this article as no datasets were generated or analysed during the current study.
